# U. S. V. M. A. Proceedings

**Published:** 1897-08

**Authors:** 


### U. S. V. M. A. PROCEEDINGS.

WITH the July number of the JOURNAL we completed the production for our subscribers of the entire proceedings of the United States Veterinary Medical Association Convention of 1896. This was secured for our readers at a great expense to the publishers of the JOURNAL. It has been reproduced in a manner by which it can be readily removed from the issues of the JOURNAL and bound in a separate volume, so that those who are not members can have a complete report of the doings of the national organization at no expense beyond the regular subscription fee of the JOURNAL. In doing this the JOURNAL has at the same time given to its readers the average quota of sixty-four pages of other matter, and thus added this report as a premium. The '96 meeting of the Association was one of the best ever held in the history of the U. S. V. M. A.,

**Section work will be on trial. Are you preparing to sustain your special line of interest at Nashville?**

and, therefore, the report of its doings was the recording of an important epoch in association achievements. It was the only complete report produced by any veterinary journal in North America, and in this is a true index of the JOURNAL'S purpose in sustaining the best interests of the veterinary profession in North America. Many of those who subscribed only for 1897 failed to receive the early portions of the proceedings, which commenced with the October number of 1896. We provided a sufficient number of the last three numbers of 1896 to fill one hundred extra orders of these numbers for those who wish to complete for reference or binding this report, at the regular price for the JOURNAL. The value of these reports increases with each year added to the association's history, and to the coming meeting at Nashville is added increased interest by the wide dissemination of the past work of this organization, and thus journalism keeps full pace in all its efforts to sustain and enlarge the usefulness and power of association circles in better doing the work of the profession's advancement.

**Representatives from States never before represented in our conventions will be with us.**

